# The bactericidal effect of two photoactivated chromophore for keratitis-corneal crosslinking protocols (standard vs. accelerated) on bacterial isolates associated with infectious keratitis in companion animals

**DOI:** 10.1186/s12917-022-03397-z

**Published:** 2022-08-17

**Authors:** Anja Suter, Sarah Schmitt, Ella Hübschke, Malwina Kowalska, Sonja Hartnack, Simon Pot

**Affiliations:** 1 Ophthalmology Section, Equine Department, Vetsuisse Faculty, University of Zurich, Zurich, Switzerland; 2 Veterinary Bacteriology Section, Institute for Food Safety and Hygiene, Vetsuisse Faculty, University of Zurich, Zurich, Switzerland; 3 Epidemiology Section, Vetsuisse Faculty, University of Zurich, Zurich, Switzerland

**Keywords:** PACK-CXL (photoactivated chromophore for keratitis—corneal crosslinking), Riboflavin, Infectious keratitis, Bacterial keratitis, Veterinary

## Abstract

**Background:**

Bacterial corneal infections are common and potentially blinding diseases in all species. As antibiotic resistance is a growing concern, alternative treatment methods are an important focus of research. Photoactivated chromophore for keratitis-corneal crosslinking (PACK-CXL) is a promising oxygen radical-mediated alternative to antibiotic treatment. The main goal of this study was to assess the anti-bactericidal efficacy on clinical bacterial isolates of the current standard and an accelerated PACK-CXL treatment protocol delivering the same energy dose (5.4 J/cm^2^).

**Methods:**

Clinical bacterial isolates from 11 dogs, five horses, one cat and one guinea pig were cultured, brought into suspension with 0.1% riboflavin and subsequently irradiated. Irradiation was performed with a 365 nm UVA light source for 30 min at 3mW/cm^2^ (standard protocol) or for 5 min at 18mW/cm^2^ (accelerated protocol), respectively. After treatment, the samples were cultured and colony forming units (CFU’s) were counted and the weighted average mean of CFU’s per μl was calculated. Results were statistically compared between treated and control samples using a linear mixed effects model.

**Results:**

Both PACK-CXL protocols demonstrated a significant bactericidal effect on all tested isolates when compared to untreated controls. No efficacy difference between the two PACK-CXL protocols was observed.

**Conclusion:**

The accelerated PACK-CXL protocol can be recommended for empirical use in the treatment of bacterial corneal infections in veterinary patients while awaiting culture results. This will facilitate immediate treatment, the delivery of higher fluence PACK-CXL treatment within a reasonable time, and minimize the required anesthetic time or even obviate the need for general anesthesia.

## Introduction

All vertebrate species can be affected by secondary bacterial corneal infections once the corneal epithelial barrier has been compromised. Opportunistic microorganisms can originate from the normal ocular flora, and take advantage of a weakened ocular surface defense system, leading to a corneal infection [1, 2]. The inflammatory response to infection activates proteolytic collagen-dissolving enzymes in the corneal stroma resulting in ‘corneal melting’, which can lead to corneal ulcer deepening, corneal perforation and loss of vision despite intensive medical therapy [1–4]. Intensive medical management, including the frequent application of topical antibiotic and anticollagenase eye drops, is the current gold standard non-surgical corneal ulcer treatment [1, 5, 6]. However, there are growing concerns regarding antibiotic resistance [7–14], which might mitigate the efficacy of medical ulcer therapy. Surgical interventions may significantly increase corneal fibrosis and lead to potentially severe vision impairment [2, 15–17]. Therefore, there is a need for the development of alternative treatment methods targeting bacterial viability and enzymatic corneal melting in corneal ulcers.

An alternative or adjunctive corneal ulcer treatment method has been proposed in the form of corneal cross-linking [18–20], utilizing UV-A light and riboflavin [18–20]. CXL is a procedure that was developed for the treatment of keratoconus in humans, in which it arrests progressive loss of structural integrity of the corneal stroma [20, 21]. Riboflavin (Vitamin B_2_) acts as a photosensitizer when exposed to UV-A light with a wavelength at one of its absorption peaks (365 nm), which results in the generation of free radicals [22–26]. This process leads to free radical-induced photochemical crosslinking and the formation of chemical bridges between protein residues (proteoglycans) and collagen fibers, and/or other molecules within the corneal stroma [21, 27–29], thus increasing the biomechanical and biochemical stability of the cornea by improving its’ resistance to enzymatic digestion [20, 21]. CXL can also lead to free radical-induced elimination of microorganisms. Riboflavin diffuses through cellular membranes and intercalates with microorganismal nucleic acids, inducing genomic damage [25, 26, 30–32] and damaging multiple targets within microorganisms [33–35]. As a result, microbial pathogens are far less likely to develop resistance to CXL than to traditional antibiotics [36–38], which is an important advantage of CXL over medical therapy.

CXL was shown to effectively arrest corneal melting and treat infectious keratitis in clinical cohort studies and in prospective trials in veterinary and human patients [39–49]. The clinical use of CXL for the treatment of corneal infections was renamed ‘photoactivated chromophore for keratitis-corneal crosslinking’ (‘PACK-CXL’) and established in human and veterinary medicine [44, 47, 50, 51]. PACK-CXL has a variable inhibitory effect on microorganisms in vitro, depending on the type of microorganism and differences in treatment protocols [23, 52–54], though it has been shown that antibiotic-resistant and non-resistant bacteria were equally sensitive to PACK-CXL [55].

A bactericidal effect has been demonstrated using standardized, non-ocular strains or single strains obtained from human patients [23, 53, 54, 56, 57]. However, genetic variability between strains and isolates could affect their susceptibility to external physical and chemical stimuli [58, 59], which could explain some of the observed variability in clinical efficacy.

According to the Bunsen–Roscoe photochemical law of reciprocity [60], the effects of any photochemical reaction (in the current context, the PACK-CXL procedure) can be maintained as long as the total energy delivered (fluence) is maintained by adapting the radiation intensity to the energy delivery time [61]. This implies that the effect of the PACK-CXL treatment should be similar for a 30 min standard irradiation of 3 mW/cm^2^ and a 5 min accelerated irradiation of 18 mW/cm^2^, provided that the total energy delivered (5.4 J/cm^2^) is identical [61, 62]. Accelerated PACK-CXL is desirable as it would shorten the duration of, or obviate the need for, general anesthesia in veterinary patients. Accelerated PACK-CXL would also allow the delivery of higher fluences, which increase the tissue-stabilizing effect [62–65], while keeping the length of treatment within reasonable limits. However, CXL-induced biomechanical stiffening of the cornea is oxygen dependent and decreases with treatment acceleration and intensity increase [66, 67]. For example, Bao et al. demonstrated that irradiation protocols of 10 min at 9mW/cm^2^ and 30 min at 3mW/cm^2^ had a similar biomechanical stiffening effect, whereas protocols of 5 min at 18mW/cm^2^ and shorter were not as effective [68, 69].

Riboflavin intercalation-induced genomic damage to microorganisms makes it plausible that the antimicrobial effect of PACK-CXL is at least partially oxygen-independent and should not be affected by shortening of the PACK-CXL procedure [70]. Indeed, Richoz et al. did not observe a difference in antimicrobial effect between accelerated (5 min, 18mW/cm^2^) and high acceleration (2,5 min, 36mW/cm^2^) standard fluence (5.4 J/cm^2^) PACK-CXL [52].

The objective of this study was to assess the antimicrobial efficacy, measured as reduction of CFU’s per µl, of standard PACK-CXL (30 min, 3mW/cm^2^) and accelerated PACK-CXL (5 min, 18mW/cm^2^), with both protocols delivering the standard fluence of 5.4 J/cm^2^. Various bacterial isolates from clinical veterinary patients with infectious keratitis were used to test for differences between isolates regarding sensitivity to PACK-CXL treatment.

## Materials and methods

### Bacterial isolates

Eighteen wild type bacterial isolates derived from veterinary patients with infectious keratitis (eleven dogs, five horses, one cat, one guinea pig), presented to the University of Zurich Veterinary Medical Teaching Hospital in 2013 and 2014, and isolated at the Section of Veterinary Bacteriology (VB), Vetsuisse Faculty, University of Zurich, were selected for use in this study.

Species identification was performed by matrix-assisted laser desorption/ionization time-of-flight mass spectrometry (MALDI TOF MS, Bruker Daltonics GmbH, Bremen, Germany) in addition to standard bacteriological procedures. All isolates were either *Staphylococcus* (*n* = 8), *Streptococcus* (*n* = 5), *Pseudomonas* (*n* = 2), *Pasteurella* (*n* = 2) or *Frederiksenia* species (*n* = 1) (Table 1), since those had previously been identified as the most commonly isolated bacterial pathogens from patients with infected corneal ulcers presented to our clinic [14] .

**Table 1:** Bacterial isolates used in the experiment

Bacterial genus	Case Number	Species	Sampled species
***Staphylococcus***	14–1547 SK2	*Staphylococcus aureus*	Horse
	15–1745 SK1	*Staphylococcus aureus*	Horse
	14–1774 SK2	*Staphylococcus epidermidis*	Dog
	15–395	*Staphylococcus epidermidis*	Dog
	15–1852 SK1	*Staphylococcus epidermidis*	Guinea Pig
	15–1913 SK5	*Staphylococcus pseudintermedius*	Dog
	15–1125 SK1	*Staphylococcus haemolyticus*	Dog
	15–1305 SK2	*Staphylococcus lentus*	Horse
***Streptococcus***	15–799 SK1	*Streptococcus equi* ssp. *zooepidemicus*	Dog
	15–1371 SK1	*Streptococcus canis*	Dog
	15–1913 SK4	*Streptococcus canis*	Dog
	15–1305 SK1	*Streptococcus equi* ssp. *zooepidemicus*	Horse
	14–1547 SK1	*Streptococcus dysgalactiae* ssp. *equisimilis*	Horse
***Pseudomonas***	15–1308 SK1	*Pseudomonas aeruginosa*	Dog
	15–1670 SK1	*Pseudomonas aeruginosa*	Dog
***Pasteurella***	15–1353 SK1	*Pasteurella multocida*	Cat
	16–110 SK1	*Pasteurella dagmatis*	Dog
***Frederiksenia***	15–1371 SK3	*Frederiksenia canicola*	Dog

*Frederiksenia canicola* was initially classified into the *Pasteurellaceae* family, a consecutive identification with MALDI-TOF resulted in this different and more accurate classification. Because it was historically included in the genus *Pasteurella*, and for a simplified overview (because both genera only had very few isolates), *Frederiksenia canicola* was counted to the family of *Pasteurellaceae* in the results section [71].

### Riboflavin solution

A 0.1% iso-osmolar riboflavin solution was used in all experiments. A 0.1% concentration was achieved by diluting 2 ml riboflavin (Vitamin B2 Streuli, Uznach, Switzerland) in 8 ml 0.9% NaCl (B. Braun Medical AG, Sempach, Switzerland) (Table 2).

**Table 2:** PACK-CXL protocol details

PACK-CXL	Standard	Accelerated
Treatment target	Bacterial suspension	
Soak time and interval	30 min, continuous: bacteria suspended in Ri/NaCl solution	
Chromophore	Riboflavin (Vitamin B2 Streuli, Uznach, Switzerland)	
Chromophore carrier	0.9% NaCl (B. Braun Medical AG, Sempach, Switzerland)	
Chromophore osmolarity	Iso-osmolar	
Chromophore concentration	0.1%	
Light source	UV-X^tm^ illumination system (version 1000), IROC, Switzerland	CCL-VARIO Cross-linking system, Peschke Trade, Switzerland
Wavelength (nm)	365	
Irradiation mode	Continuous	
Fluence (J/cm^2^)	5.4	
Intensity (mW/cm^2^)	3	18
Treatment time (minutes)	30	5

### Bacterial suspensions

Each cryopreserved isolate (Table 1) was streaked onto a fresh Columbia Blood Agar with Sheep Blood (Thermo Fisher Diagnostics AG, Pratteln, Switzerland) and incubated under aerobic conditions for 20–24 h at + 37 °C. A 0.5 McFarland suspension was then prepared from these cultures using 0.9% NaCl solution. The bacterial concentration of this suspension amounted to be 1.5 × 10^5^/μl. Three μl of this suspension were diluted 1:10 in a 0.1% riboflavin/0.9% NaCl solution (treatment groups: standard or accelerated PACK-CXL) or in a 0.9% NaCl solution (control groups: standard or accelerated C). The resulting starting suspensions had a bacterial concentration of 4.5 × 10^4^/30 μl and were used as treatment and control samples in the experiments.

### PACK-CXL and quantification

Four experimental groups were defined: “standard PACK-CXL” (0.1% riboflavin/0.9% NaCl sample, 30 min UV-A irradiation at 3mW/cm^2^), “standard Control” (0.9% NaCl sample, no irradiation, 30 min), “accelerated PACK-CXL” (0.1% riboflavin/0.9% NaCl sample, 5 min UV-A irradiation at 18mW/cm^2^) and “accelerated Control” (0.9% NaCl sample, no irradiation, 5 min).

30 μl volumes of the control (standard/accelerated Control) and therapy samples (standard/accelerated PACK-CXL) were pipetted into single wells of a 48-well-plate (Falcon® Multiwell 48 well, Corning Incorporated, Corning, USA).

The sample plates were shaken for 1 min at 500 rpm (MTS 2/4 digital, IKA, Staufen, Germany), then wrapped in aluminum foil leaving a treatment window above the therapy samples (standard or accelerated PACK-CXL) to protect the control samples from the UV irradiation and from ambient light. The wrapped plates were placed underneath the CXL-lamp at an optimal 5 cm focal distance. The UV energy output of the CXL light sources (3mW/cm^2^ and 18mW/cm^2^) was measured with the enclosed UV-light-meter. For the standard PACK-CXL protocol, the CXL treatment wells (standard PACK-CXL) were irradiated for 15 min with a UV-A device (UV-X^tm^ illumination system (version 1000), IROC, Switzerland) at 3mW/cm^2^. The plates were then placed on a plate shaker for one minute at 500 rpm and irradiated again for another 15 min. After irradiation, the plates were placed on the plate shaker for another minute. For the accelerated protocol, the PACK-CXL treatment wells (accelerated PACK-CXL) were irradiated for 5 min with a UV-A device (CCL-VARIO Cross-linking system, Peschke Trade, Switzerland) at 18mW/cm^2^. The details of the PACK-CXL procedure are listed in Table 2. After irradiation, the plates were placed on the plate shaker for one minute at 500 rpm. Subsequently, 30 μl samples of PACK-CXL-treated solution (standard or accelerated PACK-CXL) and of non-irradiated control solution (standard or accelerated Control) were retrieved from the wells, pipetted into separate Eppendorf tubes and diluted 1:10 with 0.9% NaCl, followed by serial dilutions. From the dilutions, 100 μl aliquots were plated in duplicate onto Columbia Blood Agar with Sheep Blood (Thermo Fisher Diagnostics AG, Pratteln, Switzerland). The agar plates were incubated overnight for 20–24 h at + 37 °C under aerobic conditions. The experiment was replicated twice with each isolate on different days. Duplicate agar plates containing between 15 and 300 colonies were counted and the formula below was used to calculate the weighted average mean of colony forming units per μl.

$$Conversion\;formula:\;C=\frac{\Sigma c}{n1\times1+n2\times0.1}\times d$$

C = weighted average mean of colony numbers. Σc = sum of colonies of all plates, n1 = number of plates with the lowest evaluable dilution stage, n2 = number of plates with the next higher evaluable dilution stage, d = factor of the lowest evaluable dilution stage.

### Preliminary trials: temperature and evaporation

A few technical details were evaluated in preliminary trials to optimize the experimental conditions. Temperature measurements were conducted due to our concern of inducing a significant temperature increase in the small volumes of irradiated medium, which would potentially lead to bacterial growth alteration and loss of sample volume due to evaporation. No temperature change occurred as measured with an IR thermometer (IR Thermometer Dual Laser EXTECH INSTR. 42,509, FLIR Commercial Systems Incorporated, Nashua, USA) during the 30-min irradiation of 30 μl 0.9% Natrium Chloride solution (B. Braun Medical AG, Sempach, Switzerland) with 3mW/cm^2^ irradiance. No significant fluid evaporation was detected during a 30-min irradiation with 3mW/cm^2^ irradiance as measured via fluid repipetting post PACK-CXL treatment.

### Statistical analysis

With the aim to assess if the bactericidal effect (reduction in CFU’s per µl) differed significantly between treated and control samples, bacterial genus (*Pseudomonas*, *Staphylococcus*, *Streptococcus*, *Pasteurella, Frederiksenia*) and host species (horse, dog, cat, guinea pig) a mixed model was performed in R version 4.0.5 using the packages nlme Pinheiro 2016 [72] and biostatUZH [73]. The isolate was considered as random effect. Model selection was based on the Likelihood Ratio Test and Akaike Information Criterion (AIC), with lower values of at least 2 indicating a better model fit. Subsequently, the Test for interaction according to the method described by Gail and Simon was performed to determine whether evidence for a different bactericidal effect between accelerated PACK-CXL and standard PACK-CXL existed [74]. The results for the first and second replicate were analyzed separately to avoid multiplicity, as suggested in methodological publications [75, 76]. Results from both analyses (replicate 1 and 2) are presented to ensure consistency of the results and use of all data.

## Results

**Fig. 1:**
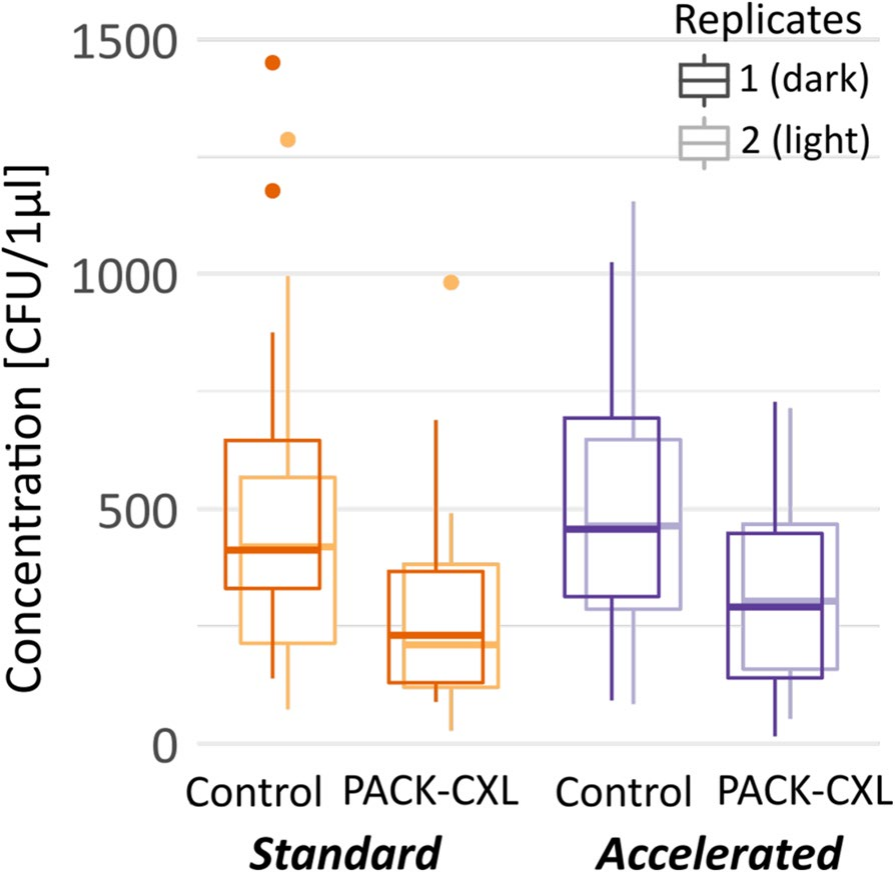
CFU’s/μl for PACK-CXL treated and untreated control samples. Bacterial concentration [CFU’s/μl] in PACK-CXL treated and untreated control groups, presented as box-plots. Horizontal thick lines represent the median, horizontal thin lines represent the 25th and 75th percentiles, dots are outliers

**Fig. 2:**
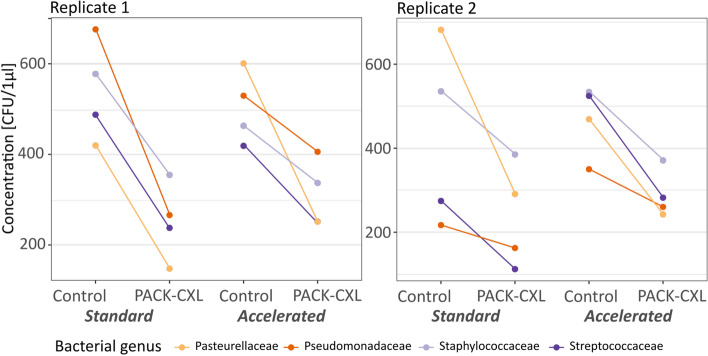
CFU’s/μl for PACK-CXL treated and untreated control samples grouped by bacterial genus or family. Bacterial concentration by bacterial genus or family presented as mean (dot) [CFU’s/µl]. Lines indicate drop in mean concentration after PACK-CXL treatment

Weighted average mean CFU’s/μl were calculated for the various experimental groups and are presented in the box plot diagram below (Fig. 1). A significant difference in CFU’s/μl between the control samples and the PACK-CXL treated samples was observed in both experimental replicates (Fig. 1, Table 3). PACK-CXL treatment led to an average reduction of -217 CFU’s/μl (*p* < 0.0013, 95% CI from -334 to -100) and -185 CFU’s/μl (*p* < 0.001, 95% CI from -305 to -66) in experimental replicates 1 and 2, respectively. CFU’s per μl for PACK-CXL treated and untreated control samples grouped by bacterial genus or family are presented in Fig. 2 and Tables 4 and 5. A statistical difference between bacterial genera or family regarding microbe reduction following PACK-CXL was not observed. Host species did not significantly influence bacterial concentrations. No evidence was found for a difference in treatment effect between the standard 30-min 3mW/cm^2^ and the accelerated 5-min 18mW/cm^2^ PACK-CXL treatment protocols (replicate 1: *p* = 0.48; replicate 2: *p* = 0.97).

**Table 3:** Bacterial concentration [CFU’s/μl] for PACK-CXL treated and untreated control samples (replicates 1 and 2)

	Standard		Accelerated		n
	Control	PACK-CXL	Control	PACK-CXL	
Replicate 1	537 ± 337 (138–1450)	278 ± 173 (89- 688)	481 ± 267 (92–1025)	307 ± 203 (15–727)	72
Replicate 2	452 ± 329 (73–1286)	269 ± 227 (27–982)	500 ± 269 (84–1155)	313 ± 181 (52–714)	72

Data presented as: mean + standard deviation (minimum – maximum); n = sample size

**Table 4:** Bacterial concentration [CFU’s/µl] for PACK-CXL treated and untreated control samples grouped by bacterial genus or family (replicate 1)

	Standard		Accelerated		n
	Control	PACK-CXL	Control	PACK-CXL	
*Pasteurellaceae*	420 ± 221 (280–675)	148 ± 77 (89–234)	601 ± 368 (370–1025)	252 ± 170 (143–448)	3
*Pseudomonas*	676 ± 281 (477–875)	266 ± 154 (157 = 375)	530 ± 270 (339–720)	406 ± 230 (243–568)	2
*Staphylococcus*	578 ± 379 (291–1450)	355 ± 171 (114–688)	463 ± 207 (148–720)	337 ± 208 (123–727)	8
*Streptococcus*	488 ± 400 (138–1177)	238 ± 202 (91–588)	418 ± 354 (92–818)	252 ± 244 (15–5566)	5

Data presented as: mean ± standard deviation, (minimum – maximum); n = sample size

**Table 5:** Bacterial concentration [CFU’s/µl] for PACK-CXL treated and untreated control samples grouped by bacterial genus or family (replicate 2)

	Standard		Accelerated		n
	Control	PACK-CXL	Control	PACK-CXL	
*Pasteurellaceae*	682 ± 401 (230–995)	291 ± 229 (41–491)	469 ± 344 (269–866)	242 ± 206 (107–480)	3
*Pseudomonas*	217 ± 98 (148–286)	163 ± 72 (111–214)	350 ± 32 (327–373)	260 ± 69 (211–309)	2
*Staphylococcus*	535 ± 342 (209–1286)	385 ± 265 (145–282)	534 ± 325 (84–1155)	371 ± 222 (52–714)	8
*Streptococcus*	275 ± 228 (73–564)	112 ± 73 (27–207)	525 ± 222 (243–859)	282 ± 132 (114–473)	5

Data presented as: mean ± standard deviation, (minimum – maximum); n = sample size

## Discussion

A variety of studies have demonstrated that PACK-CXL is a potentially valuable adjunctive or alternative therapy for the treatment of infectious keratitis in both human and veterinary patients [43, 47, 48, 51, 77–80]. The bactericidal effect of accelerated (2.5 and 5 min) PACK-CXL protocols delivering a standard fluence of 5.4 J/cm^2^ against sequenced reference strains has previously been established in the laboratory study [52]. Our study confirms the lack of difference in bactericidal efficacy of standard (30 min) and accelerated (5 min) PACK-CXL protocols delivering a standard fluence of 5.4 J/cm^2^ against „wild type “bacterial isolates previously isolated from veterinary patients affected with infectious keratitis.

High fluence protocols are attractive for clinical use as they may improve PACK-CXL treatment effects. Various authors have demonstrated an increase in antibacterial efficacy with PACK-CXL fluence increases. Bacterial killing rates increased from 50–60% with a standard 5.4 J/cm^2^ fluence to 85–100% with triple fluences of 15–16.2 J/cm^2^, at which level a plateau was reached regarding antibacterial efficacy [63, 81, 82]. Our results support the use of accelerated PACK-CXL protocols in the veterinary clinic and would thus facilitate the delivery of higher PACK-CXL fluences within a reasonable treatment time.

Despite some heterogeneity in microbe reduction following PACK-CXL between different genera/family of bacteria (Fig. 2), no statistical differences between bacterial genera/family were observed. Differences in susceptibility to PACK-CXL between bacterial genera have been observed previously [22, 55, 82]. However, apart from one study by Martins et al. [23], all published literature in which susceptibility to PACK-CXL was compared between different bacterial strains, types and genera supports equal PACK-CXL susceptibility for *Staphylococcus*, *Streptococcus* and *Pseudomonas* spp. [52, 53, 55, 81, 82]. Furthermore, various authors reported that antibiotic resistant bacteria were as susceptible to PACK-CXL treatment as non-resistant bacteria [23, 53, 55].

Most of the studies referenced above [52, 55, 81, 82] had a similar experimental design to our study. In those, bacterial suspensions of various volumes with a maximum fluid column height of 300 μm (Makdoumi et al.: 400 μm) [55] or 150–200 μm corneal lamellae [52] were subjected to PACK-CXL protocols of different fluences (5.4, 7.2 and 15 J/cm^2^) and accelerations (30–2,5 min). As such, neither fluence nor acceleration seems to affect bacterial genus-dependent susceptibility to PACK-CXL. Using a different experimental design, Martins et al. and Schrier et al. performed PACK-CXL irradiation of agar plates, which might explain the different outcome reported by Martins et al., who observed a lower susceptibility to PACK-CXL in *Pseudomonas* spp. compared to *Staphylococcus* and *Streptococcus* spp. [23, 53].

Since the main bacterial genera of clinical importance (*Staphylococcus*, *Streptococcus* and *Pseudomonas* spp.), as well as antibiotic resistant and non-resistant isolates seem equally susceptible to PACK-CXL, a single antibacterial PACK-CXL protocol can probably be used indiscriminately in the clinic, without the need to tailor PACK-CXL protocols to target organisms. However, real differences in susceptibility to PACK-CXL between bacterial genera/family cannot be excluded in our study as this study lacks the statistical power needed to detect such differences. Therefore, further sufficiently powered studies (with greater sample sizes of bacterial isolates) are needed to draw definitive conclusions regarding differences in sensitivity to PACK-CXL between bacterial genera or species.

The effects of currently used routine CXL protocols reach up to a depth of 300 μm in corneal tissues [83, 84], and sometimes less, depending on species or CXL protocol adaptation [85, 86]. We therefore decided on an experimental design with a 30 μl bacterial suspension in 10 mm wells. The 30 μl volume was sufficiently large to be handled without major pipetting losses, and sufficiently small to create a maximum fluid column height of approximately 300 μm, likely allowing sufficient UV-A energy delivery throughout the entire fluid volume. In preliminary experiments, we used a design with microkeratome-cut porcine corneal lamellae of defined thickness and optimal reproducibility to create a setup closely resembling the real-life situation. These lamellae were placed onto a cell culture plate and barely covered with a 30 μl bacterial suspension, similar to the design used by Richoz et al. [52]. However, we decided not to use this experimental design in our main study since no obvious differences in results were observed between the experimental protocols with and without corneal lamellae. Most importantly, the protocol involving corneal lamellae yielded less reproducible results in our hands.

Furthermore, we decided to analyze both replicates separately. This allowed us to link control samples to their respective PACK-CXL treated samples in the statistical model.

One limitation of this study is that the in vitro experimental conditions with transparent fluid columns of defined height are very different from the typical clinical situation in an infected cornea where tissue edema and inflammatory cell infiltrates cause corneal thickening and opacification.

The 300 μm CXL treatment effect depth is unlikely to be sufficient in infected patient corneas which are thickened due to tissue edema and where opaque inflammatory cell infiltrates decrease UVA penetration. Tissue thickening would place microorganisms in the deeper layers of the cornea out of reach of PACK-CXL and tissue opacities would further shield them from the UVA irradiation and bactericidal effects of PACK-CXL. A complete eradication of resident pathogens from infected corneas therefore seems unlikely. Indeed, Kling et al. demonstrated a reduced bactericidal effect when irradiating 40 μl bacterial suspensions with a 1000 μm fluid column height, compared to 11 μl volumes with a ~ 300 μm fluid column height [81]. They concluded that this likely occurred because of a lower UVA intensity in the deeper sections of these 1000 μm fluid columns and a higher absolute number of surviving bacteria in the 40 μl samples.

Such critical discrepancies between the in vitro and in vivo situations can prevent the in vivo translation of in vitro findings and hamper the implementation of novel therapies in the clinic. Clinicians and scientists need to be aware of this potential disconnect and attempt to develop relevant disease models, e.g. ex vivo corneal models of infection [87, 88].

Another limitation of this study is that it was not designed to detect differences in susceptibility to PACK-CXL between bacterial genera or family, which is why the study was underpowered to detect such differences. The clinical effectiveness of the tested PACK-CXL protocols can therefore not be guaranteed.

## Conclusion

Our study provides evidence that accelerated (5 min) and standard (30 min) PACK-CXL protocols delivering a standard fluence of 5.4 J/cm^2^ do not differ in bactericidal efficacy, with no observed differences in susceptibility to PACK-CXL between bacterial genera or family. Accelerated PACK-CXL can therefore be recommended for empiric use in the treatment of bacterial corneal infections in veterinary patients while awaiting culture results. This will facilitate immediate treatment, the delivery of higher fluence PACK-CXL treatment within a reasonable time, and minimize the required anesthetic time or even obviate the need for general anesthesia.

## Data Availability

The datasets used and/or analysed during the current study are available from the corresponding author on reasonable request.

## Abbreviations

PACK-CXL: Photoactivated chromophore for keratitis-corneal crosslinking
CFU’s: Colony forming units
UV: Ultra violet
UV-A: Ultra violet A
CXL: Corneal Crosslinking
MALDI-TOF MS: Matrix-assisted laser desorption/ionization time-of-flight mass spectrometry
